# The Meso-Level in Quality Improvement: Perspectives From a Maternal-Neonatal Health Partnership in South Africa

**DOI:** 10.34172/ijhpm.2024.7948

**Published:** 2024-05-21

**Authors:** Helen Schneider, Solange Mianda

**Affiliations:** School of Public Health & SAMRC Health Services to Systems Research Unit, University of the Western Cape, Cape Town, South Africa.

**Keywords:** Quality Improvement, Meso-Level, Stewardship, District Health System, South Africa, Maternal Health, Neonatal Health

## Abstract

**Background:** Sustained implementation of facility-level quality improvement (QI) processes, such as plan-do-study-act cycles, requires enabling meso-level environments and supportive macro-level policies and strategies. Although this is well recognised, there is little systematic empirical evidence on roles and capacities, especially at the immediate meso-level of the system, that sustain QI strategies at the frontline.

**Methods:** In this paper we report on qualitative research to characterize the elements of a quality and outcome-oriented meso-level, focused on sub/district health systems (DHSs), conducted within a multi-level initiative to improve maternal-newborn health (MNH) in three provinces of South Africa. Drawing on the embedded experience and tacit knowledge of core project partners, obtained through in-depth interviews (39) and project documentation, we analysed thematically the roles, capacities and systems required at the meso-level for sustained QI, and experiences with strengthening the meso-level.

**Results:** Meso-level QI roles identified included establishing and supporting QI systems and strengthening delivery networks. We propose three elements of system capacity as enabling these meso-level roles: (1) leadership stability and capacity, (2) the presence of formal mechanisms to coordinate service delivery processes at sub-district and district levels (including governance, referral and outreach systems), and (3) responsive district support systems (including quality oriented human resource, information, and emergency medical services [EMS] management), embedded within supportive relational eco-systems and appropriate decision-space. While respondents reported successes with system strengthening, overall, the meso-level was regarded as poorly oriented to and even disabling of quality at the frontline.

**Conclusion:** We argue for a more explicit orientation to quality and outcomes as an essential district and sub-district function (which we refer to as meso-level stewardship), requiring appropriate structures, processes, and capacities.

## Background

**Key Messages**

**Implications for policy makers**

- The roles and capacity of the meso-level (such as the district or sub-district) in health systems are key to sustained quality improvement (QI) and achieving health outcomes.
- However, meso-level systems are often poorly oriented to supporting QI and addressing health outcomes.
- A quality-oriented meso-level requires leadership and decision-making power, mechanisms of service delivery coordination and review, locally negotiated referral and outreach strategies and responsive district human resource, information and resource systems.

**Implications for the public**

Encouraging people and communities to utilise health facilities has little value if the quality of care provided falls short of what the World Health Organization (WHO) defines as “care that is safe, effective, people-centred, timely, efficient, equitable and integrated.” Strategies have been developed to improve quality in health facilities and are being implemented across the globe. However, to be anchored in everyday health service routines and practices, these strategies need appropriate support from higher levels of the system. Drawing on experiences with improving the quality and outcomes of maternal and newborn care in South Africa, this paper describes the roles, structures and capacities required at sub-district and district levels to implement and sustain quality of care in health facilities. We refer to this function as the “meso-level stewardship of quality.”

Quality improvement (QI) is a burgeoning field of research and interest in low- and middle-income countries (LMICs). Ideas such as the plan-do-study-act (PDSA) cycles, learning collaboratives, continuous QI, and the array of associated technologies and tools^1,2^ have become part of everyday discourse in health systems across the globe. Changing frontline practice is seen as the “last mile” of achieving health outcomes for Sustainable Development Goal priorities^3^; and a 2018 Lancet Global Health Commission placed quality in health systems on the universal health coverage (UHC) agenda.^4^ The promise of UHC—financial risk protection and increased coverage—means little if services are of poor quality, mistrusted by communities and under-utilized^5^; or if increased utilization does not result in improved outcomes and acceptability.^4^ The World Health Organization (WHO) defines quality as “care that is safe, effective, people-centred, timely, efficient, equitable and integrated.”^6^

 Drawing on this emerging global consensus, and building on long standing initiatives to strengthen maternal, newborn, and child health in South Africa’s health system, including District Clinical Specialist Teams^7^ and maternal and perinatal death reviews,^8^ the Mphatlalatsane Project (hereafter referred to as “the project”) was a South African initiative which aimed to reduce maternal and neonatal mortality by 50% through QI strategies in districts of three provinces (Mpumalanga, Limpopo, and Eastern Cape).^9^ This 5-year (2018-2022) initiative was steered by a national partnership of governmental, non-governmental and academic players, and implemented a range of facility-based QI interventions, including PDSA cycles, training, audit, and patient support in the target districts.

 In baseline interviews with partners conducted as part of the project evaluation, the limits of facility-based QI interventions were well recognised. Concurring with the observation made in the Lancet Commission that “fixes at the micro-level (ie, health-care provider or clinic) alone are unlikely to alter the underlying performance of the whole system,”^4^ respondents emphasized the sub-district and district (generically referred to as meso-level) as the immediate contexts shaping facility-level processes. The project designers envisaged district-level strategies such as strengthened referral and outreach systems, but ultimately, in the context of the COVID-19 disruptions, these received limited attention. In the words of a senior manager, *“that meso-level, the district, is missing from this equation.”*

 With respect to defining a meso-level role, the WHO recently launched a multilevel ‘Quality Toolkit’ outlining strategies spanning national, sub-national/district, facility and community levels include regulation, measurement/audit, and supervision systems.^10^ In addition, WHO proposed “quality standards” for maternal-newborn health (MNH) that include components such as information systems and human resources.^11^ These guidance documents, however, do not address the background system capacity required to implement strategies.

 The meso-level is increasingly part of research designs for QI in health systems. Examples include engaging facility or district managers to co-design, steer and support change processes,^12^ or to address quality “bottlenecks”^13^; district peer “learning networks”^14^; and performance-based financing mechanisms.^15^ However, this intervention research mostly approaches the meso-level as a support to the micro-level, rather than being its central focus, or alternatively, as the proximal organisational and system context explaining variations in micro-level performance.^16-18^

 There is relatively little systematic and situated empirical evidence on the meso-level role, or intervention research on QI that privileges action at this level. In Belgium, Gray^19^ explored the meso-level functions of sense-making and distributed leadership within professional networks as enablers of integrated care systems. In a similar vein, establishing and nurturing professional networks across hospitals played a key role in improving newborn care in Kenya,^20^ and regional collaborative networks supported maternal care in Tanzania.^21^ We previously described how a combination of formal/hierarchical and informal/networked district governance enabled MNH in districts of two South African provinces.^22,23^ The similarities between these experiences, despite vastly different settings, provide useful starting points for thinking about the meso-level, and suggest that lesson learning across jurisdictions is possible.

 We report on qualitative research seeking to answer the question: what are the roles and capacities of the meso-level necessary for QI and how can these be strengthened in South Africa? We locate the meso-level between the provincial/national macro- and facility micro-levels, which in South Africa (as in many other countries), corresponds to the formal governance structures of the district and sub-district health system (DHS). Drawing on the embedded experience and tacit knowledge of project partners involved in MNH, we define the roles, capacities and enabling systems required at the meso-level for quality, but which may be “missing from the equation.” Through this, we aim to shift the focus from QI technologies/interventions and scale up strategies^24^ to the systems in which these interventions are embedded.^14^

###  Setting

 South Africa has a plural health system, in which the public sector is the majority provider (82.6% of the population), alongside a resource-rich and insurance based private sector.^25^ Public primary healthcare (PHC) is widely accessible, comprehensive in nature and free at the point of use; and is linked to a district hospital network which also provides free maternal and child healthcare embedded within a DHS. The DHS is an important social safety net, and South Africa is an outlier amongst LMICs in having low out-of-pocket payments for healthcare (<6% in 2019).^26^ Day to day health sector decision-making is devolved to nine provincial governments in a three-tier political system. These provinces are demarcated into 52 districts and 226 sub-districts, coterminous with the boundaries of local government. The DHS is enshrined in the National Health Act of 2003 as the most decentralized building block of South Africa’s health system. Although the sub-district is not formalised in legislation, in terms of population size and service delivery profile, it best approximates the WHO concept of a DHS. As part of wider reforms under the umbrella of National Health Insurance,^27^ initiatives are underway to strengthen the decentralized governance capacity of both district and sub-DHSs and to create a unified system of financing and provision of PHC.

## Methods

 A qualitative descriptive study, based on in-depth interviews was conducted. The interviews were conducted in the early phases of a larger mixed methods evaluation of the Mphatlalatsane Project (hereafter referred to as the “project”), that included a prospective evaluation of implementation processes, maternal and neonatal quality of care and mortality outcomes. Over an 18-month period (February 2020 to August 2021), a total of 22 baseline and follow-up interviews explored the processes and contexts of implementation with project designers and partners (total of 10 respondents, 1-4 interviews per respondent) (Table 1). The study sample was constituted of members of the Mphatlalatsane Project Management Committee, which included the key National Department of Health (NDoH) actors responsible for design and oversight of the project and the various project partners involved in implementing activities in the districts. These partners were based in university research units and non-governmental organisations (local and international). All had considerable prior experience with strengthening district level MNH services in South Africa.

**Table 1:** Interviews Conducted by Stakeholder Groups (n = 13, 39 Interviews)

**Stakeholder Group**	**Baseline**	**Follow-up**
NDoH (n = 3)	3	5
IP (n = 7)	7	7
QI advisor debriefings (QI advisor) (n = 3)	17	

Abbreviations: NDoH, National Department of Health; IP, implementing partners; QI, quality improvement.

 Interviews were conducted virtually, mostly with individuals and a few jointly, lasting between 45 minutes to one hour. Guided by previous research,^22,23^ the interviews covered a range of macro and meso-level themes, including perceptions on (interview schedule in Supplementary file 1):

- The development and evolution of the partnership
- The nature and extent of leadership commitment to maternal and neonatal health in the three provinces
- The functioning of structures and processes for improved maternal and neonatal health
- Availability of resources for maternal and neonatal health

In follow-up interviews the authors specifically probed for views on the meso-level—roles, capacities, enablers/constraints, and change strategies—which in large part form the basis of this analysis. Interviews were conducted at the height of the COVID-19 pandemic when project activities were frequently stopped or interrupted. We thus asked participants to reflect on their wider knowledge base and experience beyond the project sites, and the examples provided do not necessarily refer to the Mphatlalatsane Project only.

 In addition to these interviews, we participated in Project Management Committee meetings (n = 13) and drew on regular debriefing sessions (n = 17) held with the three “QI advisers” who were coordinating implementation of QI interventions in the project districts. These debriefings mostly focused on micro-level progress (the pre-dominant focus of the project), but also provided occasional reports of action at higher levels of the system (eg, facilitating district referral pathways).

 The interviews and debriefings were audio-recorded and transcribed and analysed thematically. In a first step of immersion, the co-authors independently read documents and transcripts and, in some instances, listened to the audio files. From this first step we defined an initial set of domains as follows:

- Making the case for the meso-level
- Meso-level roles and capacities
- Meso-level decision-space required
- Supporting change

The co-authors then manually extracted data in spread sheets using these domains, assigned codes to the data, grouped these into themes, and in an iterative process, reframed the domains into the three main themes reported in the findings: roles, system capacities that enable roles (with three sub-themes: leadership capacity, area-based service coordination, and responsive district systems), and meso-level change strategies.

 We also scanned several dozen project documents issued over the period, including project plans, presentations, reports, and project management committee minutes, to triangulate/verify these constructs. A technical report of the findings was circulated to interviewees for validation and presented at project meetings before the final version was made publicly available.^28^ A framework developed from this analysis guided the subsequent phase of data collection with provincial and district managers.

## Results

 We start with the views of respondents on the roles required of district and sub-DHSs to improve and sustain quality and health outcomes. We then describe the system capacities which make possible these roles and conclude with partner views on meso-level change processes.

###  Roles

 Interviewees proposed a number of meso-level roles in advancing quality of care and health outcomes. The meso-level mediated the macro- and micro-level spheres to translate policy into implementation; drove and encouraged frontline providers to innovate; introduced QI strategies and systems; coordinated and aligned actors and initiatives; and managed key service delivery systems, notably referral and outreach support (Table 2). All these roles require a meso-level that has the capacity to act autonomously and is far more than a “post box,” conveying instructions from above and transmitting reports from below back up the system.

**Table 2:** Roles of Meso-Level Actors in Maternal and Neonatal Quality of Care and Outcomes

**Role**	**Details**	**Quotes**
Mediate between macro and micro levels	Translate policy into implementation	*“They are well placed not to be too high up such as national and provincial, but at the same time, they also have direct access to the facilities and hospitals in a way so that they are able to drive from the bottom and from the top to be able to deliver on the healthcare outcomes” *(IP).
Enable and drive action	Encourage frontline providers to innovate and implement; prevent disablement	*“The people who make decisions, these are district managers and facility CEOs, these are key decision makers and for any improvement work or activity to even begin, these too need to become the sponsors or drivers of that improvement activity” *(IP). *“At the frontline, there are a lot of people who are working as hard as they can, to do their jobs as they should, but the system itself seems to be disabling rather than enabling” *(NDoH). *“What happens at facility ends up being paralyzed by the multiple layers above it, all of which appear, to many people at facility level to be placed there precisely to stop them from doing things” *(NDoH).
Establish QI systems	Introduce QI projects, develop systems and ensure coverage	*“The district clinical specialist introduced a number of quality improvement projects. And they had a system already going, where they were doing some quality improvements with the whole district with … [the] hospital’s drainage area”* (IP).
Coordinate and align actors and activities	Alignment and coordination of prorammes (“indicators”) and quality initiatives, addressing fragmentation	*“The problem with the district offices and in some places even replicated in subdistrict offices is that we have one manager per indicator, … the management layers have ballooned with people who are responsible for reporting essentially, on a single indicator. And that has fragmented the system”* (NDoH). *“There was not much coordination, each clinic or district had different quality improvement programs which were not linking to one and other” *(IP).
Manage key service delivery and other systems	Referral and outreach and supportive systems, and clinical governance	*“The DMT [district management team] is mainly looking at systems but it also has clinical governance [roles], where they look at the real clinical care… The governance structure that they put together now, they, it’s opened the in-reach and the out-reach so you refer to a person, and you can have contact with the person whether a consultant or a specialist prior to actually referring the patient…” *(NDoH).

Abbreviations: NDoH, National Department of Health; IP, implementing partners; QI, quality improvement; CEO, chief executive officer.

 These roles imply capabilities in local problem solving, maximising efficiencies, learning from experience and resilience. While the meso-level was generally viewed as *“disabling rather than enabling,”* respondents had witnessed positive illustrations of such capabilities. One was a successful initiative, in the early phases of the project, to shift normal maternal deliveries away from an overburdened hospital to appropriately staffed and resourced community health centres. The initiative involved serial meetings with key stakeholders (hospital and district managers, emergency medical services [EMS] and referral facilities), presenting evidence through data and analyses, and jointly identifying bottlenecks and negotiating solutions. The results were “*impressive, and that was just facilitating communications and making everyone understand and everyone pulling in the same direction”* (QI advisor). In another district, access to specialised drugs for newborn care was decentralised to limit unwarranted referrals: *“the district hospitals would say that we were told by [our] pharmacy that a level 1 hospital is not supposed to have these drugs and that’s why we refer all the patients to you guys [regional hospital]… and on the spot, they had a meeting with the [L2] pharmacists and managers and [they] said, listen, we can stock that drug so you don’t have to send the babies to us. We will order enough for all of our district hospitals and then you just ask from our pharmacy, … and keep the baby here” *(IP).

 An underlying theme, frequently referred to in interviews was the phenomenon of *agency*: *“the single most important objective of this project, is to work out how to give people back their agency” *(NDoH). Agency was viewed as both the willingness and the freedom to take action: *“you do have situations where you have very motivated front liners who don’t really care about what is happening up there… you would have some facilities that would say, we want this, we want to do it with or without the permission of an HOD [Head of Department], and then you get to another facility where they say if we don’t have a letter we can’t talk to you” *(NDoH). Agency in decision-making came from a combination of courage and skills: *“Those are communication issues which they can actually deal with locally at the district, and if you had a proper manager, that could happen, but that’s often lacking. And the courage to make decisions is also lacking so they just continue as they are” *(IP).

###  System Capacities That Enable Meso-Level Roles

 From interviews and project documents we formulated three themes of local health system capacity as key to enabling meso-level roles in MNH: leadership capacity, ability to coordinate service delivery processes at sub-district and district levels, and responsive district support and systems.

####  Leadership Capacity

 Meso-level capacity rested fundamentally on the presence of stable and skilled leaders at all levels of the health system: “*you can get enthusiastic people on the ground, and they can improve the situation in their hospital, and they can have good ideas… but to take it beyond that is almost impossible because of the lack of capacity and stability in the middle management”* (IP). Interviewees characterised middle management, especially in hospitals, as chronically unstable, with many in acting positions. *“In just about every place there have been a number of managers over the time and people keep coming and going and every time you have to start fresh … particularly at the management level”* (IP). Fractious labour relations and politicised leadership appointment systems were regarded as underlying problems.

 Although stable leadership was a necessary condition, the buy-in and commitment of leaders were also key to mobilising the micro-level *“because [when] they’ve bonded with the project … then they allow the people on the ground to continue with the implementation”* (NDoH). In one project site “*the provincial involvement and the district involvement and the whole catchment area [enabled] people to take ownership and … to get involved” *(NDoH).

 Finally, meso-level managers also needed leadership and management skills alluded to earlier: ability to motivate others and foster team-work, negotiate upwards, ability to see the “big picture” and apply systems thinking, analyse problems to steer change processes, and the confidence and resilience to make and follow through on decisions. In reality, professionals were often placed in managerial positions without preparation or support and lacked “*the experience necessary to manage and … the skills and clinical knowledge to be able to affect any change”* (IP). These included *“simple things”* like effective and efficient meeting practices.

 One interviewee described a contradiction between the “*highly experienced and learned” *frontline clinical championswithout powerand a weak meso-level without the capacity to exercise power: *“we have created this system that disempowers progressively as you go down the ranks. You get to a point where you’re at the bottom, you’re just at the bottom of the pile. And even doctors and specialists at the bottom of the pile are scared of the authorities” *(NDoH).

####  Area-Based Service Coordination

 Recognising the continuum of care and “many hands”^29^ involved in achieving MNH outcomes, a key design feature of the Mphatlalatsane Project, codified in project documents, was to support the coordination of services in a service delivery “wedge.” In this wedge, the basic unit of service delivery is the district hospital and surrounding clinics and community-based services. This unit or catchment area maps onto, even if not exactly, to demarcated sub-district boundaries, and in turn, to a referral, regional hospital, which the district authority sometimes managed. Collectively these formed the project “wedge.” The various service delivery units, interfaced—not necessarily in a straightforward manner—with hierarchical lines of authority to the sub-district and district.

 Coordination of service delivery in the “wedge” required:

- Governance structures focused on quality and outcomes that connected clinical, managerial and programmatic players, sometimes across sub-district and district boundaries;
- Referral systems between units, negotiated with EMS; and
- Systems of skills development, specialist clinical outreach, and support.

Interviewees pointed out a challenge in the absence of management structures and coordination processes at sub-district level, and the resulting lack of coordination between PHC and district hospitals, which reported in separate lines to the district. While “forums” existed between the two, these were generally ad hoc, informal arrangements and relationships often portrayed as antagonistic involving “blame games” and some degree of “resistance.” The interface between the district and regional hospitals was also considered weak: “*regional hospitals actually work as islands, in isolation outside their catchment environment” *(NDoH).

 Through the project, implementing partners (IP) sought to introduce a mechanism of sub-district governance focused on quality and outcomes, which coordinated local line, clinical, information, EMS and programme managers (spanning PHC, hospital, and sub-district). As one interviewee remarked: *“If managers are not accountable, then nothing is going to change, and it goes all the way down” *(IP). The sub-district mechanism would build on and extend existing maternal and perinatal death review processes, and combine functions normally associated with clinical governance (clinical audit, guidelines, and training), with public health, health programme management and the managerial functions of sub-district and district structures (See Box 1). A corresponding monitoring and response mechanism in the larger service delivery unit (the district) would coordinate regional hospitals and managers from the sub-district and district. The district coordinating mechanism would feed into with district planning and quarterly performance review processes, and through these shape decision-making on resource allocation, service redesign and referral systems development.

**Box 1. Functions of the Sub-district Governance Mechanism^28^**

- “Undertake real-time reviews of morbidity and mortality data, identifying the links between actions at PHC and community level and outcomes at the hospital level.
- Develop appropriate responsive action – whether skills development or system action.
- Assign roles and responsibilities to actors and hold them accountable for decisions and plans.
- Identify appropriate responses required at higher levels of the system (district and province).
- Develop communications channels (formal and informal) for day-to-day problem solving.”

--------------------

Abbreviation: PHC, primary healthcare

Service delivery coordination also required functioning patient referral systems. Although policies on referral systems in a unified public health system are straight forward,^30^ interviewees reflected on the many complexities and tensions inherent in implementing MNH referral systems. These tensions related, amongst others, to a lack of consensus on whether normal maternal deliveries should happen in PHC facilities, an over-burdening of district hospitals, the uneven distribution of skilled staff and unreliable EMS. These were compounded by the lack of data on referral processes.

 A functioning MNH referral system rests on locally negotiated referral processes (a description of such a process is provided in Supplementary file 2), enabled by informal relational ecosystems for day-to-day problem solving. As one interviewee reflected:

 “*We are coming from the situation where people just refer, [you have a piece of] paper and refer to an unknown, unnamed, unidentified person. But now, the governance structure that they put together now, … so you refer to a person, and you can have contact with the person whether they be a consultant or a specialist prior to actually referring the patient, so actually the cooperation is much better”* (NDoH).

 Advances in technology can support relationships, with remote communication having “leap frogged” over the COVID-19 waves and local WhatsApp groups ubiquitous. IP had also experimented with a South African developed mobile phone application specifically developed to support referral processes, called “Vula.” The Vula App enables confidential communication between clinicians on individual patients, and also simultaneously collates data on the referral system which to date had been *“completely non-existent”* (IP).

 The third element of service delivery coordination is clinical outreach. There are currently two main models in South Africa: dedicated District Clinical Specialist Teams, and the geographical service area model, integrated into the hospital system where clinicians provide support to the next level in a cascade. As one interviewee pointed out *“it does not matter whether it is the Western Cape process *[geographical service area model]* or the district clinical specialist process, but you need the skilled people, you need organized outreach and a systematic way along a clearly defined process”* (IP). Structured outreach programmes included the “safe Caesar” package, “essential steps for managing midwife and obstetric emergencies fire drills,” “facility assessment tools,” and the “helping babies breathe” training package, which together have been credited for the declining maternal and neonatal mortality in South Africa.^31^ Outreach systems were also considered key to mobilising the necessary equipment and broker infrastructural development with higher levels of the system.

 Clinical outreach thus combined personal and team mentorship, skills development, specialised patient care and advocacy roles, in which regional hospitals played a key role. As explained by a project designer: *“regional hospitals have specialists, and these specialists … are meant to oversee the entire clinical operations in their wedge, in their catchment area. And what we wanted to see happen here is that the clinical leadership actually takes ownership and accountability for all clinical processes – case management, case referral, down referral, out referral, clinical support for them, clinical support meaning outreach support for clinical care and governance, reviewing data as a unit, responding to data as a unit” *(NDoH).

 Underpinning all three elements of service delivery coordination were efforts to challenge the siloed mindsets of system actors, and to develop an appreciation of the whole system (an example of this in relation to outreach is provided in the Supplementary file 3).

####  Responsive District Support and Systems 

 The system capacities for MNH quality and outcomes outlined above are themselves embedded within core district level systems. These include the traditional health system building blocks (eg, human resource, information and supply chain management systems, infrastructure development) or system “hardware” and its responsiveness to quality and outcomes, and the “software” of capacity and cultures of decision-making at this level.

 District systems that centre quality and responsiveness would ensure, for example, the availability and appropriate distribution of advanced midwives, MNH skills development plans, the design of referral systems, procurement of ambulances and strengthening leadership capacity. However, as interviewees pointed out, the provincial and national decision-making and accountability cultures surrounding the meso-level players encouraged an orientation toward upward compliance rather than downward service delivery support:

*“… the rules that we put in place to protect the system against things like fraud, like the PFMA *[Public Finance Management Act]* actually land up disabling us from being able to do anything. Because we are so hedged with regulations, rules, and provisos … it’s more important to follow rules than it is to deliver services” *(NDoH).

 A compliance orientation has a freezing effect on responsive action at the meso-level.

*“…you go all the way down to sub-district level and all the way up the system … you seem to only find people who can tell you what you can’t do but not people who can answer as to how you can do things. It has become a very risk averse system…” *(NDoH)and;

*“… if you do nothing, you can’t get blamed, whereas if you are proactive and do something, you run the risk of doing something wrong and getting hammered, so, it is actually safer to do nothing” *(NDoH).

 Meso-level “decision-space” – the product of delegations in authority, sub-district and district capacity to exercise this authority and aligned accountabilities^32^ – is effectively narrow. Not only are *“district-level managers … not able to implement any initiative without the approval of the provincial managers,” *they fear punitive action if seen to be “breaking the rules” by taking initiative, and lack the capacity to claim the spaces that are available to them.

###  Meso-Level Change Strategies

 Interviewees gave examples of promising strategies to shift the meso-level away from a dominant compliance culture. As one pointed out *“it is possible to give people back their agency, but I don’t think it happens just automatically, no” *(NDoH). These strategies have been alluded to in the examples provided above.

 A common feature of positive experiences was investment in nurturing relationships, whether convening new spaces of interaction between levels of the system vertically, or in strengthening horizontal networks. For example:

*“There was a lot of animosity between the district hospitals and the clinics, they hated each other. So we still needed to fix that relationship. So now they view each other as one unit… it is not a hospital and PHCs, they actually call the hospitals their mum. We have developed a WhatsApp group where all our managers and champions are on that group and they encourage each other all the time” *(IP).

 Interviewees emphasised that creating support for change required patience and engagement over time. *“…it takes a lot of motivation, a lot of hand holding” *(IP).Processes of co-production or co-design with local actors built support for change, mobilised their tacit knowledge, and allowed for context-specific approaches. *“…we learned quite a lot from our previous experience that if we are going to implement any changes or any ideas…, there is a need to actually locally adapt this to the context that we are working in. And if that’s not being done with teams at the provincial level, teams at the district level, teams at the sub-district level, and then teams at the facility level - then we will not see any gain”* (IP).

## Discussion

 Based on the grounded experiences and insights of partners in a MNH initiative, we have described the enabling roles and system capacities required of district and sub-DHSs for QI. Respondents referred to roles typically associated with middle managers in health systems,^33^ while also highlighting the importance of deliberate strategies and systems at the meso-level to enable facility level QI. In general, the meso-level was considered as “missing from the equation” in supporting MNH quality, and as having weak capacity and limited power. However, respondents believed that strengthening the meso-level was feasible, and gave examples of successful initiatives that drew on their long standing embedded system understanding.

 While the need for “whole system” perspectives^2^ and multi-level action^10,14^ on quality is now accepted, there is relatively little written specifically on meso-level system capacity. English et al,^34^ in their analysis of the interaction between micro- and meso-level factors in QI in Kenya, provide insight into the general mechanisms of the meso-level. They refer to three local “resource systems”—material, skills and relational systems—and five “motive forces” of change (eg, leadership, goal alignment, responsive planning, empowerment, and learning).

 This analysis reports similar mechanisms, but locates these within district and sub-district governance contexts. This paper has sought to systematise the roles and systems capacity of the meso-level for MNH quality and outcomes as a core (but often weak or missing) district and sub-district function, requiring appropriate structures, processes, capacities, and decision-space. The meso-level role in quality is best described as a form of decentralised *stewardship* (denoting its collaborative nature), which is not reducible to clinical governance or formal district management planning and evaluation, but which seeks to bring these together in new mechanisms of coordination and governance. Such systems are described in the emerging literature on care networks^21^ and on quality collaboratives in high income country contexts.^35^

 Drawing on our empirical findings, we propose a model of meso-level capacity for quality and outcomes involving the following interacting elements (represented in Figure):

- Leadership capacity, including stability, skills and motivation;
- Area-based service coordination through appropriate governance, accountability, referral and outreach systems; and
- Responsive district systems, oriented towards quality.

**Figure:**
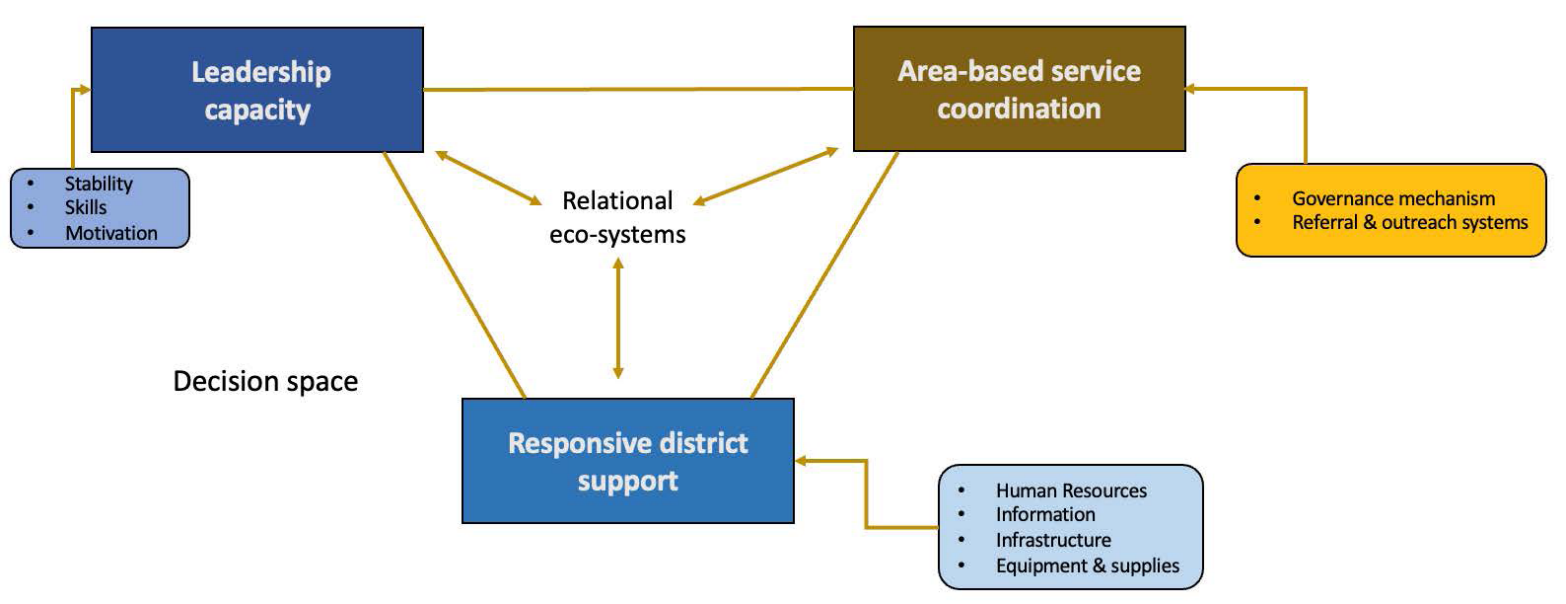
Elements of Meso-Level Capacity for Quality.

 These are embedded within supportive informal and formal relational ecosystems, and appropriate decision-space.

 Guidance on the meso-level role in QI complements MNH clinical guidelines and standards and contributes to thinking on strengthening the performance of sub-district and DHSs. This is especially relevant as UHC-inspired reforms are being implemented (eg, the National Health Insurance proposals) with these decentralised system elements as their building blocks.

 The model can also inform research on QI, and as indicated, was applied in further phases of the Mphatlalatsane Project evaluation. It provides a way to approach the analysis of the meso-level in a directed fashion, in contrast to more all-encompassing frameworks of context^17,36^; it also enables consideration of how facility level interventions simultaneously interact with and shape local system contexts in a dynamic fashion.^37^

 Recognising the crucial role of the meso-level should prompt wider reflections on macro-level factors that enable or undermine the capacity of the meso-level to fulfil its roles. In South Africa, audit and surveillance systems in the field of MNH have proliferated, responding to global maternal and neonatal mortality targets set in the Millennium and then Sustainable Development Goals. Unfortunately, these accountability mechanisms have tended to reward upward reporting rather than local problem solving and learning.^38^ Decision-making remains centralised in provincial and national spheres, and district and sub-district authority and capacity for resource allocation and problem solving on quality-related issues is limited.^39^

 Others have called for a “governance reset” in which “frontline governance is strengthened to support QI” and there is “adequate and appropriate authority delegations to district managers.”^40^ This requires “reconfiguring and creating greater coherence in roles and structures of district and sub-DHSs, accompanied by widened decision-space”^28^ and a systematic approach to leadership and management development at these levels. The insights gained from the Mphatlalatsane Project offer an approach to thinking about local system contexts that enable quality.

 The analysis presented has limitations. Firstly, it is centred on the perspectives of national decision-makers and IP. Despite the considerable local experience of these partners, they are not the views of district and sub-district actors themselves. However, these were obtained in subsequent phases of the Mphatlalatsane Project evaluation and confirmed the findings of this paper,^41^ while offering further nuance, including for example on the importance of informal networks and relationships.^14,19^ Secondly, examining the meso-level through the lens of MNH foregrounds aspects of local health systems, including the management of emergencies, in ways that are different to the quality challenges of care for long term conditions such as HIV or non-communicable diseases. The meso-level roles in the latter may emphasize dimensions such as service integration, continuity of care and social support. Related to this, the framework does not address the systems of patient and public participation and accountability,^42^ which we previously highlighted as a more general weakness of governance in South Africa’s health system.^22^

## Conclusion

 Drawing on insights from the field of MNH, this paper has argued for the necessity, and outlined the elements of a meso-level role in healthcare quality and outcomes as an integral component of decentralised governance through DHSs. However, the meso-level in South Africa is regarded as still weakly oriented towards, and at times actively disabling of, quality. Addressing this requires not only strengthening meso-level structures, systems and processes, but reorienting systems at all levels.

## Acknowledgements

 The evaluation team is deeply grateful to the Mphatlalatsane project designers and initiators in the NDoH and amongst the implementing partners for so readily sharing their insights, experiences and documentation. Arrie Odendaal and Terusha Chetty of the Mphatlalatsane evaluation team provided valuable feedback on the manuscript. A technical report was compiled prior to the writing of this manuscript entitled: Schneider H, Mianda S. *District and sub-district stewarship of quality and health outcomes: roles, systems and strategies (Briefing Document).* Cape Town: University of the Western Cape; 2022. Available at: https://repository.uwc.ac.za/handle/10566/7498.

## Ethical issues

 The study was approved by the Biomedical Ethics Research Committee of the University the Western Cape (BM19/10/16), and the SAMRC Ethics Committee (EC019-11/2019). All interviews proceeded with the consent of participants, with attention paid to anonymity in the presentation of findings. Quotes are referenced broadly (as outlined in Table 1), but where attribution is unavoidable to those familiar with the setting (such as a specific experience) we sought permission to include this before publishing the material.

## Competing interests

 Authors declare that they have no competing interests.

## Funding

 This work was supported by the South African Department of Science and Innovation/National Research Foundation South African Research Chair’s Initiative (grant number 98918); and by the ELMA Philanthropies via the South African Medical Research Council to UWC (grant number 46241). The funders played no part in the design and conduct of the study, data collection, data management, data analysis and interpretation, preparation, review or approval of the manuscript.

## Supplementary files

Supplementary file 1. Interview schedule.

Supplementary file 2. Description of a Participatory Workshop to Develop Obstetric Referral System.

Supplementary file 3. Example of a Process To Develop Systems Thinking for Neonatal Outreach.
