# Supplementary material for: The Meso-Level in Quality Improvement: Perspectives From a Maternal-Neonatal Health Partnership in South Africa
Source: Int J Health Policy Manag. 2024 May 21;13:7948. doi: 10.34172/ijhpm.2024.7948 (PMC11270612; doi:10.34172/ijhpm.2024.7948)
Supplement: Supplementary file 1 — Interview schedule. [file ijhpm-13-7948-s001.pdf]

Article title: The Meso-Level in Quality Improvement: Perspectives From a Maternal-Neonatal Health Partnership in South Africa

Journal name: International Journal of Health Policy and Management (IJHPM)

Authors' information: Helen Schneider*, Solange Mianda

School of Public Health & SAMRC Health Services to Systems Research Unit, University of the Western Cape, Cape Town, South Africa.

***Correspondence to:** Helen Schneider; Email: hschneider@uwc.ac.za

Citation: Schneider H, Mianda S. The meso-level in quality improvement: perspectives from a maternal-neonatal health partnership in South Africa. Int J Health Policy Manag. 2024;13:7948. doi:10.34172/ijhpm.2024.7948

Supplementary file 1. Interview schedule

Mphatlalatsane evaluation: Objective 4a baseline interview meso and macro level stakeholders

Overview

1. From your point of view, what is Mphatlalatsane? What do you see as the overall purpose of Mphatlalatsane and what are its key features?
2. Can you please tell me when and how it was introduced and how it has unfolded since (i.e. its implementation)?
3. What has been your role in the design and/or implementation of Mphatlalatsane?
4. Who have been the key people involved in the planning and implementation of Mphatlalatsane?
5. What do you see as the key achievements of Mphatlalatsane so far? What do you think has made possible these achievements?
    a. (If not addressed) Has Mphatlalatsane changed the way district and facility actors do their work? If yes, how?
6. What do you see as the key challenges of Mphatlalatsane so far and the reasons for these challenges?
7. In your opinion, do you think Mphatlalatsane was implemented the way it was intended? Have there been any unexpected effects (positive or negative) that have emerged as the result of the implementation of Mphatlalatsane?

Governance structures and processes

8. Can you please describe, if any, the existing structures for decision making related to the quality and outcomes of MNH in the three intervention areas? What is your view of the functioning of these bodies?
9. How does Mphatlalatsane relate to these structures, has it shaped their functioning in any way, or introduced new structures and processes?

Leadership, cooperation and coordination

10. How would you describe participation and buy-in to Mphatlalatsane in the three catchment areas? Amongst partners?
11. Are all the relevant actors at sub-district, district, regional hospital and provincial level part of the implementation process of Mphatlalatsane?
    a. Do the managers, clinicians and information officers all equally own and drive the initiative?
12. In your opinion who are the official or unofficial champions driving the implementation process of Mphatlalatsane at sub-district, district, regional hospital and area level?
13. Do you think that Mphatlalatsane has influenced how actors interact with each other, within teams (e.g. medical and nursing staff), between units (e.g. maternity and neonatal units), and between levels of care (e.g. PHC and district hospitals) and governance (e.g. sub-district and district)?
    a. If so, how have relationships and communication (formal and informal) been affected?
14. When people identify problems at sub-district, district or regional hospital levels that need to be addressed at another level, what action can and do they take?

Knowledge and information

15. Can you please tell me about data management and data use at sub-district, district, regional level? Are there mechanisms in place for continuous data collection, aggregation, analysis and feedback to frontline staff, managers and leaders?
16. How has Mphatlalatsane influenced the use of data?

Resource flows

17. Are there mechanisms to ensure the provision of resources and support in response to problems identified?
18. Are there mechanisms to ensure that key equipment is available or procured, and routinely checked to ensure functionality, and reported for action at different levels?
19. What happens where there are stockouts of essential medications or supplies at sub-district, district or regional level?

Referral relationships

20. Are criteria for referral of mother or newborn clearly defined and understood by all clinical staff at referring and referral facilities across the catchment area?
21. What are the mechanisms for referral information exchange between key providers at referring and referral facilities?
22. What is the nature of relationships between health care providers at different levels and EMS services?
23. Has Mphatlalatsane affected the way referral happens between levels? Protocols, relationships, communication and response times?

Sustainability

24. What is your view on the sustainability of Mphatlatsane? Will it survive once the external facilitation is no longer there?

Additional observations

25. Do you have any additional observations or insights to share on Mphatlalatsane or the evaluation of Mphatlalatsane?
