# Supplementary material for: The Meso-Level in Quality Improvement: Perspectives From a Maternal-Neonatal Health Partnership in South Africa
Source: Int J Health Policy Manag. 2024 May 21;13:7948. doi: 10.34172/ijhpm.2024.7948 (PMC11270612; doi:10.34172/ijhpm.2024.7948)
Supplement: Supplementary file 2 — Description of a Participatory Workshop to Develop Obstetric Referral System. [file ijhpm-13-7948-s002.pdf]

Article title: The Meso-Level in Quality Improvement: Perspectives From a Maternal-Neonatal Health Partnership in South Africa

Journal name: International Journal of Health Policy and Management (IJHPM)

Authors' information: Helen Schneider*, Solange Mianda

School of Public Health & SAMRC Health Services to Systems Research Unit, University of the Western Cape, Cape Town, South Africa.

***Correspondence to:** Helen Schneider; Email: hschneider@uwc.ac.za

Citation: Schneider H, Mianda S. The meso-level in quality improvement: perspectives from a maternal-neonatal health partnership in South Africa. Int J Health Policy Manag. 2024;13:7948. doi:10.34172/ijhpm.2024.7948

Supplementary file 2. Description of a Participatory Workshop to Develop Obstetric Referral System

"…we were using four scenarios as a workshop to improve communication and management of 1

cases. EMS were very keen to get into, so we had buyThis all related to the ESMOE programme, which provinces bought into and obviously -in from the management… We were able to run

workshops where we had some of the managers and some of the people from EMS, and we asked for people from each district. …we [then] went to a few districts and they had the EMS people from that district, plus some ambulance people. We had the CEOs and key clinician of the hospital and we had midwives from the clinics, and we had someone from the tertiary hospital. Then we put them in their various places, so the EMS sat together and they had to have a station commander, because if you refer anyone, then the midwife at the clinic has to phone the station

commander and then … there's a person that receives the call [at the call centre] and then they transmit the call.

So for example, there was one [scenario] where there was a sixteen-year-old who was brought in by her parents. Was fitting at home and is obviously pregnant, and that's at a clinic. So, then the clinic had to say what they would do, then how would they refer. So, then they had to go through the process of phoning the call centre. The call centre then dispatches an ambulance. And then the clinic, what they would do for the patient before getting into the ambulance, what the ambulance people would do while the woman is in the ambulance. Then communication between the tertiary hospital and what they were told and what information. So, we did roleplay of that whole thing… a whole lot of problems were brought up. So, for example, the EMS is not allowed to administer magnesium sulphate or anti-hypertensives. The EMS personnel are not allowed to do vaginal examinations or bimanual compression of the uterus or someone who's got postpartum haemorrhage.

And they had to … accept that the major barriers are not money, the major barriers are that people feel disempowered to implement the simple things that would make a difference." (IP)
