# Supplementary material for: The Meso-Level in Quality Improvement: Perspectives From a Maternal-Neonatal Health Partnership in South Africa
Source: Int J Health Policy Manag. 2024 May 21;13:7948. doi: 10.34172/ijhpm.2024.7948 (PMC11270612; doi:10.34172/ijhpm.2024.7948)
Supplement: Supplementary file 3 — Example of a Process To Develop Systems Thinking for Neonatal Outreach. [file ijhpm-13-7948-s003.pdf]

Article title: The Meso-Level in Quality Improvement: Perspectives From a Maternal-Neonatal Health Partnership in South Africa

Journal name: International Journal of Health Policy and Management (IJHPM)

Authors' information: Helen Schneider*, Solange Mianda

School of Public Health & SAMRC Health Services to Systems Research Unit, University of the Western Cape, Cape Town, South Africa.

***Correspondence to:** Helen Schneider; Email: hschneider@uwc.ac.za

Citation: Schneider H, Mianda S. The meso-level in quality improvement: perspectives from a maternal-neonatal health partnership in South Africa. Int J Health Policy Manag. 2024;13:7948. doi:10.34172/ijhpm.2024.7948

Supplementary file 3. Example of a Process To Develop Systems Thinking for Neonatal Outreach

"When the DCSTs were disbanded, we went to the [tertiary level] specialists, [saying] this is the new model [of outreach]. They looked at us as though we were crazy that we were saying that they must go and do what? What about the patients in their hospital? So they just didn't get the concept that problems at the PHC become your district hospital problems. Problems at the district hospital become your regional hospital problems. Regional hospitals automatically become your tertiary hospital problems.

Our first thing was that we had to get the group together. We had to come up with terms of reference for them to say, guys, this is how we're going to function as a group, these are our roles and responsibility as a group. Then the first port of call we said, okay, give us a status update of newborn care in your district. The first time we did it, our regional hospital champions will tell you about their problems in their regional hospital, in their ward. So then we'd say, okay, but that's not your district, what's happening in the whole district. They couldn't tell us. So we [introduced them] to the tool called FAST [and asked them to] go find out in your district hospitals what's going on, so when you are asked what is happening with newborn care in your district, you're talking about your entire district and not just your little unit in your hospital.

It was the biggest eye-opener for them when they went out to their district hospitals. They actually solved 80% … of the bottlenecks on the spot in their district hospitals. We accompanied some of them… in the Mphatlalatsane catchment sites, where those regional hospital champions solved problems on the spot, long-standing bottlenecks that were causing babies to die, they solved them on the spot. So they are now starting to understand how the whole system works, that they are clinicians, yes, but they are also paediatricians or specialists for the entire district. And their problems in their facility will never go away until they solve the problems in the entire district at the lower levels … it's been an eye-opener." (IP)
